# Comparative *in vivo* PET imaging of silica microparticles: shape-dependent blood circulation and short-term biodistribution

**DOI:** 10.1039/d5dt00412h

**Published:** 2025-10-08

**Authors:** Jan Grzelak, Martí Gich, Rafael T. M. de Rosales, Anna Roig, Juan Pellico

**Affiliations:** a Institute of Materials Science of Barcelona (ICMAB-CSIC), Campus of the UAB 08193 Bellaterra Spain jpellico@icmab.es; b School of Biomedical Engineering & Imaging Sciences, King's College London, St Thomas’ Hospital London SE1 7EH UK

## Abstract

Particles at the nanometric–micrometric interface hold promise by combining the drug-loading capacity of microparticles with the systemic benefits of nanoparticles. We assess the *in vivo* behaviour in mice *via* PET imaging of silica rods and spheres (∼1 μm), showing different biodistributions thus highlighting the potential for shape-dependent targeting.

## Abstract 2

We compare the *in vivo* behaviour in mice *via* PET imaging of silica rods and spheres (∼1 μm) after intravenous administration, showing different biodistributions and highlighting the potential for shape-dependent targeting.

## Graphical abstract

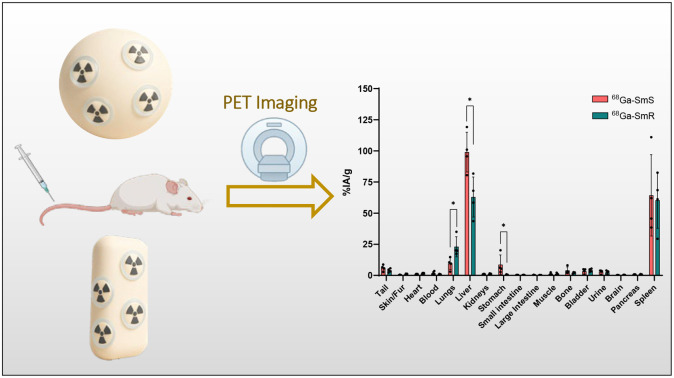

## Introduction

Inorganic particles offer numerous applications in the biomedical field, including imaging, therapy, sensing, and regenerative medicine.^1–3^ While particles between 5–300 nm are commonly used in nanomedicine for their superior biocompatibility, tissue penetration, and blood pharmacokinetics,^4,5^ microparticles between 5–100 μm have shown unparalleled drug loading capacity with distinct advantages for sustained drug release.^6^ However, the use of microparticles remains limited to non-intravenous applications due to the risk of rapid pulmonary embolization.^7,8^ Although lung entrapment is beneficial for specific applications, it is a significant concern for the broader use of particles in biomedicine, raising safety issues and demanding strict control over the physicochemical properties of the particles and administration doses to ensure suitable biodistribution and minimal patient risk. In that sense, inorganic particles at the nanometric–micrometric interface (500 nm–3 μm) have been scarcely studied but hold significant potential to achieve drug-loading capacities comparable to conventional microparticles while preserving the favourable systemic response of nanoparticles following intravenous administration. Therefore, understanding the biodistribution of inorganic particles in the submicron–micron size interface would address longstanding concerns regarding lung embolization and unlock their potential for intravenous biomedical applications. Moreover, when addressing medical uses of the particles, the relevance of the shape can be as crucial as that of the size.^9,10^ Yet, relatively few works have studied the influence of particle shape on biodistribution at early stages, and in particular, particles of distinct aspect ratios but of similar dimensions, something never explored for particles in the 1 μm size range.^11–13^

Positron emission tomography (PET) is a nuclear imaging technique that provides three-dimensional, whole-body quantitative information on biological processes using compounds radiolabelled with a positron emitter.^14^ It yields functional information with high sensitivity, making it widely used for evaluating the biodistribution and pharmacokinetics of nano/micro-formulations.^15^ PET has been employed to assess the *in vivo* fate of polystyrene microplastics, common environmental pollutants, following oral administration or inhalation, showcasing the potential of the technique to study the *in vivo* behaviour of microparticles.^16,17^ Despite the proven capability of PET to provide a comprehensive understanding of the *in vivo* fate of particles, its application to inorganic particles in the size range proposed in this work has been limited.^18^

To support the use of silica particles at the nanometric–micrometric interface for intravenous biomedical applications, we leveraged the high sensitivity and quantitative capabilities of PET imaging to assess the pharmacokinetics and biodistribution of two silica particles, spherical and rod-shaped, both approximately 1 μm in size. Silica nanoparticles have demonstrated favourable biocompatibility in both preclinical studies and clinical trials.^19,20^ These particles typically undergo hydrolytic degradation *via* the hepatic–biliary route. However, their circulation time and elimination pathways can vary considerably depending on factors such as the route of administration, particle size and shape, and surface chemistry.^21^ While their behaviour at the nanoscale is well reported, much less is known about their *in vivo* fate at the nanometric-to-micrometric interface. Here, 1 μm silica particles were radiolabelled with the positron emitter Ga-68 (^68^Ga, *t*_1/2_ = 67.8 min), the radiochemical properties were evaluated and the *in vivo* behaviour was analysed at 30 and 180 min post-intravenous administration (IV) *via* the tail vein, providing a quantitative assessment of their circulation in blood and short-term biodistribution.

## Materials and methods

All chemicals were purchased from Sigma Aldrich. Radio thin-layer chromatography (radio-TLC) was developed on Agilent Technologies glass microfibre chromatography paper impregnated with silicic acid and analysed using a Lablogic Flow-count TLC scanner and a BioScan B-FC-3200 photomultiplier tube (PMT) detector using Laura software. The ITLC mobile phase was composed of 0.175 M citric acid and 0.325 M trisodium citrate in water unless stated otherwise. Radioactive samples were measured using a Capintec CRC-25R (Capintec) or an LKB Wallac 1282 Compugamma CS (PerkinElmer) for which data were collected using EdenTerm software. PET/CT images were acquired using a NanoPET/CT scanner (Mediso), reconstructed using Nucline v.0.21 software, and images were analysed using VivoQuant software (version 3.5, InviCRO).

### Synthesis of silica microparticles with spherical (SmS) and rod shapes (SmR)

The synthesis of the particles was carried out as previously reported.^22,23^ For SmS, two solutions were prepared prior to the synthesis. Solution 1 contained 19.0 mmol tetraethyl orthosilicate (TEOS) dissolved in 33.3 mL of EtOH, while solution 2 consisted of 0.23 mmol KCl mixed with 9 mL of ammonia, 65 mL of EtOH, and 6.75 mL of H_2_O. Solution 2 was heated at 50 °C and stirred at 300 rpm for 15 min. Following this, solution 1 was added dropwise to solution 2 at a 0.2 mL min^−1^ rate. The resulting particles were purified by centrifugation at 18 300*g* for 3 min and washed five times with EtOH. The SmS particles were finally dried under vacuum.

For SmR, 2 g of pluronic P123 were dissolved in 95 mL of 1.7 M HCl. Then, the temperature was increased to 40 °C, and the mixture was stirred at 700 rpm for 3 h. Afterward, 4.2 g of TEOS was added dropwise to the solution, and the stirring was halted after 5 min, allowing the reaction to proceed under static conditions for 24 h. Then, the mixture was placed in an oven at 80 °C for an additional 24 h; the SmR were filtered and dried at 55 °C overnight. The surfactant was removed by washing the product in EtOH for 24 to 40 cycles using a Soxhlet extractor. Finally, the material was calcined in air at 550 °C for 5 h.

### [^68^Ga]GaCl_3_

Gallium-68 was eluted as [^68^Ga]GaCl_3_ from an Eckert and Ziegler ^68^Ge/^68^Ga generator in ultrapure HCl (4 ml, 0.1 M, ABX).

### Radiolabelling of SmS and SmR with [^68^Ga]GaCl_3_

The silica particles, SmS or SmR, were resuspended at 1 mg mL^−1^ in 0.5 M HEPES buffer (pH 4.9, to avoid Ga precipitation). Then, 500 μL of the solution was added into a reaction tube containing 500 μL of 0.5 M HEPES buffer (pH 4.9) before the addition of 500 μL of the [^68^Ga]GaCl_3_ elution (80–120 MBq). Reactions were conducted at 90 °C for 20 min. Afterwards, the radiolabelled particles, ^68^GaSmS or ^68^GaSmR, were purified by centrifugation at 15 300*g* for 3 min. The particles were washed three more times with phosphate buffered saline (PBS 1×) and collected by centrifugation. Finally, the particles were resuspended at 0.5 mg mL^−1^ in PBS 1× for further analysis and *in vivo* experiments.

### Radiochemical properties of ^68^GaSmS and ^68^GaSmR

The radiolabelling yield (RLY) was calculated by comparing the amount of radioactivity in the particles and the supernatants after the washing steps during purification. The radiochemical purity (RCP) was calculated by radio-TLC. For that, 2 μL of the purified particles were spotted in the chromatography strip and eluted with a solution of 0.175 M citric acid and 0.325 M trisodium citrate. Under these conditions, the radiolabelled particles remain in the origin (*R*_f_ = 0) whereas the unbound ^68^Ga^3+^ elutes to the front (*R*_f_ = 1). The amount of radioactivity in the origin and front of the chromatography strip was measured using a Lablogic Flow-count TLC scanner and compared to calculate the RCP. Finally, 200 μL of the radiolabelled particles were incubated with 400 μL of human serum at 37 °C, and 200 μL aliquots were taken at 1, 2 and 3 h to evaluate the radiochemical stability (RCS). The aliquots were centrifuged at 5000*g* for 5 min, and the amount of radioactivity in the precipitate and supernatant was measured. The RCS was calculated by comparing the amount of radioactivity in the supernatant (proteins of the serum) and the precipitate (radiolabelled particles).

### *In vivo* blood circulation

Blood samples were withdrawn at 5, 10, 20, 30, 60 and 180 min after the intravenous injection of 1.5 ± 0.5 MBq of ^68^GaSmS or ^68^GaSmR at 0.5 mg mL^−1^ in 100 μl of PBS. The radioactivity was measured in a gamma counter (LKB Wallac 1282 Compugamma CS) and the data, decay-corrected, expressed as percentage injected activity (activity in blood/total activity injected) per gram of blood (%IA per g).

### *In vivo* PET/CT imaging

Animal imaging studies were ethically handled in accordance with the Animals (Scientific Procedures) Act 1986 (ASPA) UK Home Office regulations governing animal experimentation with local approval from King's College London Animal Welfare and Ethics Review Body. *In vivo* imaging was conducted in healthy 8-week-old BALB/c mice. Animals were anaesthetized with isoflurane (2–3% in oxygen), cannulated, and placed on the scanner bed under anaesthesia. The bed was heated to 37 °C by internal air flow to keep the animal at normal body temperature, and the respiration rate was monitored and maintained at 60–80 breaths per min throughout the scan. 1.5 ± 0.5 MBq of ^68^GaSmS or ^68^GaSmR at 0.5 mg mL^−1^ in 100 μl of PBS were intravenously injected in the tail vein. PET acquisition was carried out 30 min after the administration of the particles and recorded for 20 min (1 : 5 coincidence mode; 5 ns coincidence time window). Then, a semicircular CT scan was performed after the PET acquisition. PET/CT images were reconstructed using Tera-Tomo 3D reconstruction (400–600 keV energy window, 1 : 5 coincidence mode, 4 iterations and 6 subsets) at a voxel size of 0.4 × 0.4 × 0.4 mm^3^ and corrected for attenuation, scatter and decay. PET/CT images were analysed using VivoQuant software.

### *Ex vivo* biodistribution

Uptake in different organs was evaluated by gamma counting. After the *in vivo* PET/CT imaging, animals were culled by cervical dislocation and organs were excised and weighed for radioactivity counting in a gamma counter (LKB Wallac 1282 Compugamma CS). Data were expressed as percentage injected activity (activity in the organ/total activity injected) per gram of tissue (%IA per g).

### Statistics and reproducibility

For quantitative analysis, four biological replicates were analysed for each experiment. Comparison between ^68^GaSmS and ^68^GaSmR was carried out by Student's *t*-test. A *P* value <0.05 was considered statistically significant.

## Results and discussion

### Microparticles of different shapes

Previously described silica microparticles with spherical (SmS) and rod (SmR) shapes were used for further radiolabelling reactions with gallium-68 (^68^Ga).^22,23^ As reported, both types of particles have a size of ∼1 μm with a diameter of 0.95 ± 0.05 μm and a *Z*-potential of −41 ± 3 mV for SmS and a length of 1.4 ± 0.3 μm, the aspect ratio ∼5 and a *Z*-potential of −38 ± 5 mV for SmR. These particles were selected due to their similarity in size, surface charge, colloidal stability and biocompatibility, allowing for a rigorous evaluation of the shape-dependent *in vivo* pharmacokinetics and biodistribution of silica microparticles.^24^

### Radiolabelling with ^68^Ga

**Fig. 1:**
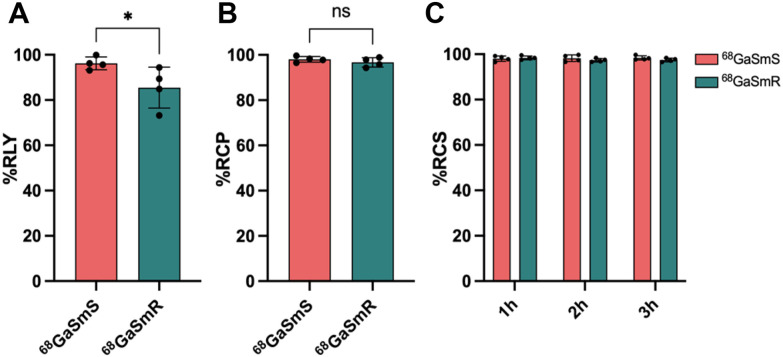
Radiochemical properties of ^68^GaSmS and ^68^GaSmR. A. Radiolabelling yield (RLY) for ^68^GaSmS and ^68^GaSmR after the incubation of the particles at 90 °C for 20 min with ^68^GaCl_3_ (*n* = 4, *P* < 0.05, two-tailed Student's *t*-test). B. Radiochemical purity (RCP) of ^68^GaSmS and ^68^GaSmR measured by radio-thin layer chromatography (radio-TLC) after the purification of the samples (*n* = 4, *P* > 0.05, two-tailed Student's *t*-test). C. Radiochemical stability (RCS) of ^68^GaSmS and ^68^GaSmR at 1 h, 2 h and 3 h after the incubation of the samples with human serum at 37 °C (*n* = 4). Data are represented as mean ± s.d.

The particles were radiolabelled with ^68^Ga using a chemical adsorption strategy. This method has been previously studied in detail, revealing a strong reaction affinity between the silanol groups of the silica and oxophilic cations such as ^68^Ga^3+^.^25^ This high affinity makes it a preferred strategy for radiolabelling silica-based materials, offering both simplicity and excellent RCS of the resulting radiolabelled particles. The combination of our particles with [^68^Ga]GaCl_3_ provided high radiolabelling yields (RLY) of 96 ± 3% for ^68^GaSmS and 86 ± 11% for ^68^GaSmR (Fig. 1A). Although the difference in RLY between particle shapes was unexpected, it might be attributed to the slightly smaller size of SmS, which results in a higher number of particles than SmR at the same concentration. Radiochemical purity (RCP) was then evaluated using radio-thin layer chromatography (radio-TLC) to ensure the absence of other radioactive species besides the radiolabelled particles. The radiolabelled samples exhibited high RCP values of 98 ± 2% for ^68^GaSmS and 97 ± 2% for ^68^GaSmR (Fig. 1B). This is crucial to prevent secondary signals in PET imaging, which could complicate the interpretation of results.

Lastly, we assessed the RCS of the particles, which measures the stability of the radiolabelling under physiological conditions. Both samples demonstrated excellent RCS values of 98 ± 1% for ^68^GaSmS and 97 ± 1% for ^68^GaSmR after incubation in human serum at 37 °C for 3 h (Fig. 1C). These very high stabilities indicate that the PET signal will predominantly originate from the radiolabelled particles rather than detached, unlabelled ^68^Ga. Overall, the radiolabelled particles exhibited excellent radiochemical properties, comparable to reported silica nanoparticles radiolabelled with positron emitters, confirming their suitability for further *in vivo* PET imaging experiments.^26^

### Blood circulation of ^68^GaSmS and ^68^GaSmR

**Fig. 2:**
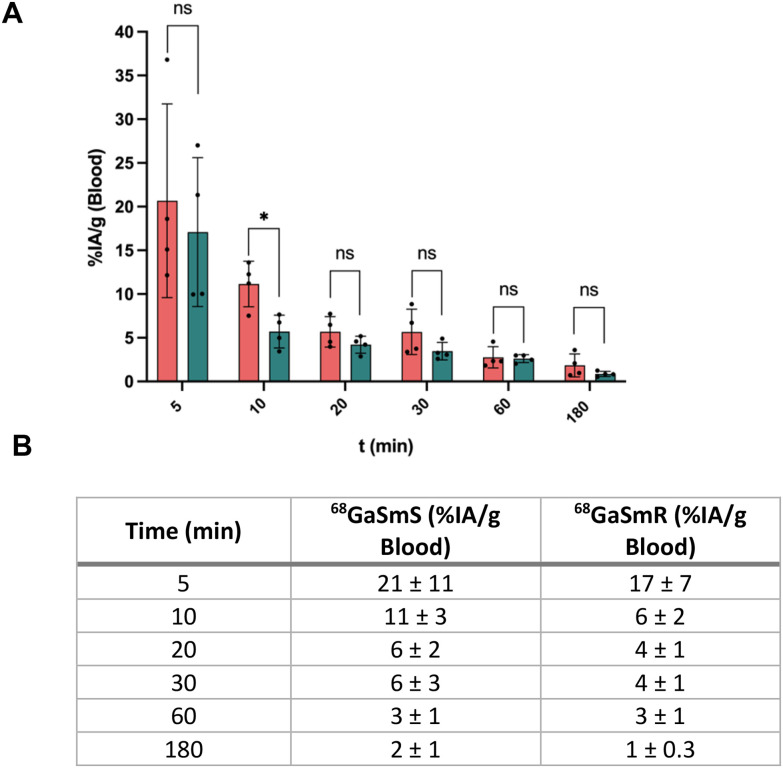
Blood circulation of ^68^GaSmS and ^68^GaSmR. A. Percentage of injected activity per gram (%IA per g) of blood at 5, 10, 20, 30, 60 and 180 min post IV administration of 1.5 ± 0.5 MBq of ^68^GaSmS and ^68^GaSmR at 0.5 mg mL^−1^ in Balb/c mice (*n* = 4, *, *P* < 0.05, two-tailed Student's *t*-test and ns, *P* > 0.05, two-tailed Student's *t*-test). B. Table depicting the values of %IA per g of blood at 5, 10, 20, 30, 60 and 180 min post IV administration. Data are represented as mean ± sd.

Blood samples were taken at different times within 3 h after IV administration of 1.5 ± 0.5 MBq of ^68^GaSmS and ^68^GaSmR at 0.5 mg mL^−1^ in 4 mice. The amount of radioactivity present in the blood was measured in a γ-counter and the data, decay-corrected, represented as a percentage of injected activity per gram (%IA per g) of blood. As expected, both particles showed a rapid elimination profile from the blood after the IV administration (Fig. 2A). The relationship between the size of particles and their clearance from the blood after IV administration is well established in nanoparticles but unknown in the size range studied here.^27^ It is known that particles smaller than 10 nm present a rapid renal clearance while particles greater than 200 nm are rapidly sequestrated by macrophages into the reticuloendothelial system organs such as the liver and spleen.^28^ Nevertheless, the circulation of particles also depends on other physicochemical parameters such as the surface charge, protein corona, the antifouling properties of the coating and mechanical properties such as the elasticity and the adhesion to cells.^29,30^ Interestingly, ^68^GaSmS showed a %IA per g in the blood of 11 ± 3% after 10 min post IV, while ^68^GaSmR presented a significantly lower value of 6 ± 2% with no significant differences for the rest of the time points (Fig. 2B).

These results indicate a slightly faster clearance of the rod-shaped particles than the spheres. Studies with nanoparticles have shown that non-spherical particles can evade phagocytes more easily than their spherical analogues and circulate for longer due to their higher anisotropy, which hinders efficient membrane wrapping by phagocytes and delays internalization.^31,32^ Specifically, silica nanorods of 150 nm have been reported to circulate for longer time than silica nanospheres of the same size.^13^ The difference in the clearance rate of ^68^GaSmR over ^68^GaSmS suggests that the shape effect in the circulation is more pronounced in particles smaller than 200 nm than in larger particles. This difference might also be attributed to better colloidal stability from the ^68^GaSmS system.

### *In vivo* PET/CT imaging and *ex vivo* biodistribution

**Fig. 3:**
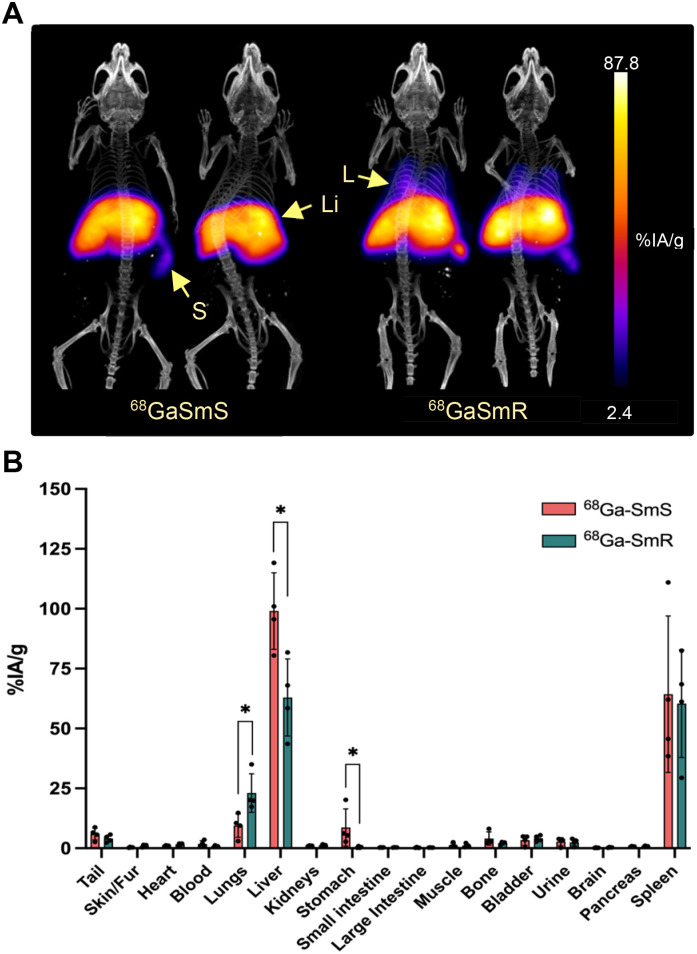
*In vivo* PET/CT imaging and *ex vivo* biodistribution. A. *In vivo* PET/CT imaging of BALB/c mice at 30 min post IV administration of 1.5 ± 0.5 MBq of ^68^GaSmS and ^68^GaSmR at 0.5 mg mL^−1^. S: spleen, Li: liver and L: lungs. Scale bar represents the percentage of injected activity per gram (%IA per g). B. *Ex vivo* biodistribution after counting the radioactivity of the excised organs in a γ-counter. Data are represented, decay-corrected, as percentage of injected activity per gram (%IA per g) 180 min after IV injection of 1.5 ± 0.5 MBq of ^68^GaSmS and ^68^GaSmR at 0.5 mg mL^−1^ (*n* = 4, *, *p* < 0.05, two-tailed Student's *t*-test).

*In vivo* PET/CT images were acquired 30 min after intravenous administration of 1.5 ± 0.5 MBq of either ^68^GaSmS and ^68^GaSmR at 0.5 mg mL^−1^ in healthy BALB/c mice. As predicted by the blood study, the *in vivo* PET/CT images showed that the particles accumulated in common elimination organs, mainly the liver and spleen, for both spherical and rod-shaped particles (Fig. 3A). Notably, ^68^GaSmR exhibited significant accumulation in the lungs, a pattern not observed with ^68^GaSmS. This difference highlights the crucial role of particle shape in biodistribution. Considering that mouse lung capillaries have been reported to be as small as 2 μm in diameter, the rod shape likely caused a portion of the particle population, particularly those with the largest sizes, to become entrapped in the lungs.

*Ex vivo* biodistribution was assessed 3 h post-injection of the particles following the same methodology as in the blood study. Consistent with the PET/CT imaging results, significant differences in *ex vivo* biodistribution were observed due to the differing shapes of the particles. The study revealed a higher lung accumulation for ^68^GaSmR, with 23 ± 9%, compared to 9 ± 6% for ^68^GaSmS (Fig. 3B). Additionally, ^68^GaSmR showed significantly lower liver uptake of 63 ± 19% in contrast to 99 ± 19% for ^68^GaSmS, while no significant differences were observed in the spleen uptake. Moreover, both particles showed a minor signal in the bladder and urine, likely corresponding to the 2–3% of free ^68^Ga^3+^ predicted by the RCS values. Overall, the results corroborate the behaviour seen in the PET/CT images, where the rod-shaped particles exhibited higher lung accumulation, leading to decreased liver uptake. These findings might have important implications for the performance of these microparticles as drug-delivery systems.

## Conclusions

Particles at the nanometric–micrometric interface are large enough to be visible under optical microscopy but small enough to potentially interact with biological systems in ways that differ from both nanoparticles and large microparticles. This study addresses the short-term *in vivo* behaviour of particles of approximately 1 μm in size with dissimilar aspect ratios upon IV administration. We successfully radiolabelled the two types of silica particles with ^68^Ga for *in vivo* evaluation using PET imaging. The straightforward radiolabelling strategy of silica provided high radiochemical yields and stabilities, facilitating successful *in vivo* blood circulation and short-term biodistribution assessments. While the particle shape did not significantly affect their radiochemical properties, it played a crucial role in their blood circulation and biodistribution. In contrast to particles of nanometric size, rod-shaped particles exhibited faster clearance than spherical ones. Moreover, the rods showed higher lung accumulation and decreased liver uptake compared to spherical particles, which were predominantly eliminated by the liver and spleen. These shape-related differences in the *in vivo* behaviour can be harnessed in future applications to target specific regions based on the intended use. Importantly, our work demonstrated the feasibility of using 1 μm size silica particles for biomedical applications in mice following IV administration at common particle concentrations. Having addressed the primary concern regarding size-related pulmonary embolization, further long-term studies would be necessary to establish a complete toxicological profile. However, given the well-documented favourable long-term safety of silica nanoparticles,^33^ we do not anticipate significant toxicity issues with our microparticles. Although smaller particle sizes are typically preferred for IV applications requiring longer circulation times, such as in cardiovascular and cancer imaging, inorganic particles at the nanometric–micrometric interface could be relevant for the delivery of higher doses of therapeutic agents to the lungs, liver, and spleen to treat and image specific conditions.

## Author contributions

J. G. conducted the synthesis of the SmR and reviewed the manuscript. M. G. supervised the experimental section and reviewed the manuscript. R. T. M. R. supervised the experimental section and reviewed the manuscript. A. R. conceived the project, supervised the experimental section and reviewed the manuscript. J. P. conceived the project, designed and conducted the experimental section and prepared the manuscript.

## Conflicts of interest

There are no conflicts to declare.

## Data Availability

The datasets used and/or analysed during the current study are available from the corresponding author on reasonable request.
